# Synthesis and investigation of dielectric ceramic nanoparticles for microstrip patch antenna applications

**DOI:** 10.1038/s41598-022-07899-6

**Published:** 2022-03-10

**Authors:** Srilali Siragam, R. S. Dubey, Lakshman Pappula, G. Satheesh Babu

**Affiliations:** 1 Department of Electronics & Communication Engineering, Swarnandhra College of Engineering and Technology, Seetharamapuram, Narsapur, A.P. India; 2 Department of ECE, KoneruLakshmaiah Education Foundation, Greenfields, Vaddeswaram, Guntur, A.P. India; 3 Department of Nanotechnology, Swarnandhra College of Engineering and Technology, Seetharamapuram, Narsapur, A.P. India; 4 Interdisciplinary Research Center, RV College of Engineering, Mysore Road, Bengaluru, KA India

**Keywords:** Electrical and electronic engineering, Nanoparticles, Materials for devices

## Abstract

Zinc aluminate (ZnAl_2_O_4_) is a well-recognized ceramic demanded in several microwave applications. Further, the addition of dielectric materials in ZnAl_2_O_4_ improved its dielectric properties, which is promising for the realization of a microstrip patch antenna. This article reports the investigation of ZnAl_2_O_4_TiO_2_ (ZAT) dielectric ceramic nanoparticles synthesized by the sol–gel process. The X-ray diffraction analysis revealed the crystalline nature of the prepared nanoparticles, with a tetragonal structure of anatase-, and rutile-TiO_2_ phases coexisting with the cubic phase of ZnAl_2_O_4_. The estimated crystallite size of the dielectric ceramic is 13.3 nm. Transmission electron microscopy (TEM) micrographs demonstrated the spherical grains with their mean diameter of 14.75 nm, whereas the selected-area electron diffraction (SAED) pattern endorsed the crystallinity of the sample. Raman measurement revealed the vibrational modes in accordance with the TiO_2_ and ZnAl_2_O_4_ compounds. The dielectric properties of the ZAT sample showed the dielectric permittivity in the range of 22.12–21.63, with its minimum loss from 0.056 to 0.041. Finally, a prototype microstrip antenna was fabricated using the prepared nanoparticles, which demonstrated a return loss of − 30.72 dB at the resonant frequency of 4.85 GHz with its bandwidth of 830 MHz.

## Introduction

Dielectric ceramic microwave (MDC) is extensively used in ultra-fast wireless networks and smart transmission systems within the millimeter wave range. The dielectric/ceramic used in millimeter wave technology must have an excellent quality factor, suitable dielectric constant, and a small resonant frequency at near-zero temperature coefficient^1,2^. In the past several decades, the microwave-based wireless communication sectors have been revolutionized by employing dielectric/ceramic materials in miniaturizing antennas with low-cost fabrication. The tailoring of the dielectric permittivity of the materials yielded their unique electrical characteristics, and therefore, found promising in miniaturizing the antenna.

Due to recent advancements in wireless communication, simple, durable, cost-effective, lightweight, low-profile patch antennas have been demanded^3^. However, due to the relatively low impedance of microstrip patch antennas, their usage in electronic equipment is limited. Numerous techniques are claimed to increase the bandwidth of microstrip patch antennas, including thick substrates, parasitic patches, and so on^4,5^. Microwave-controlled devices such as microstrip patch antennas are critical for automated high-speed cars. In recent years, considerable interest has been noticed in a novel type of microstrip patch antennas^6^. These low profile antennas are inexpensive, lightweight, and simple in processing, and they are extensively demanded in various applications, including defence and consumer products^7^. Additionally, these can operate in various wireless communication bands, including wireless local area networks, wireless fidelity, and worldwide interoperability for microwave access^8^. Further, extensive research has been focused on designing the various shapes of the microstrip patch antenna and their impact on the antenna dimension, materials used in antenna fabrication, and their corresponding performance^9^. A portable designed device based on 22 array of cylindrical dielectric antennas demonstrated the increased gain and lower-polarization. The integrated differential feeding array components were responsible for the reduced cross-polarization characteristic and enhanced antenna gain. The designed antenna exhibited − 10 dB impedance bandwidth in the frequency range from 5.78 to 5.9 GHz. The obtained antenna gains were − 30.65 dB and − 29.5 dB corresponding to the YZ and XZ planes with their resonant frequency of 5.84 GHz^10^.

Because of its unique properties, zinc aluminate (ZnAl_2_O_4_) spinel-type ceramic has become increasingly important in modern technology. This material is beneficial in various applications due to its synergetic properties, including excellent durability, moderate heat treatment, good thermal resistance, better mechanical robustness, and broad bandgap (3.8 eV)^11–13^. Additionally, this has been employed as a transparent conductor for ultraviolet radiation, detector, dielectric, and optical substances^14–17^. ZnAl_2_O_4_ has also been demanded in the telecommunications industry for resonating, filtering, and oscillating wireless fax, mobile phones, GPS, military radar systems, smart transmission systems, and satellites. The dielectric properties of ZnAl_2_O_4_ as microwave dielectric ceramic by adding TiO_2_ are studied^18–21^. They reported the potential applications of ZnAl_2_O_4_TiO_2_ in future microwave substrates and antennas. The addition of a small amount of TiO_2_ to the ZnAl_2_O_4_ resulted in an increased dielectric permittivity. Recently, the properties of ZnAl_2_O_4_ ceramic based on either composite or doped with TiO_2_, Mg_2_TiO_4_ − *x*SrTiO_3_, Co_2_TiO_4_, Mg_2_TiO_4,_ etc. are being investigated. The ZnAl_2_O_4_ based ceramic is a well-known patch material for GPS or microwave substrates. Abdullah et al. fabricated and studied the performance of the patch antenna using the nanoparticles of (1 − *x*)ZnAl_2_O_4_ − *x*SiO_2_^22^. The crystallite size of this compound was estimated to be in the range of 39.79 to 44.34 nm, along with the dielectric permittivity of 8.57. This patch antenna showed its return loss of − 14.25 dB at the resonant frequency of 3.46 GHz with its bandwidth of 60 MHz. Kim et al. studied the various dielectric ceramics having a large dielectric permittivity (ε_r_) and positive temperature coefficient of resonant frequency (τ_*f*_) values^2^. It implies that low-dielectric ceramics (< 20) are critical for frequency stability across a range of temperatures. According to Narang and Shalini et al., it is possible to improve the properties of dielectric ceramics by incorporating an appropriate material and modifying the synthesis approach^23^. A study conducted by Kumar et al. revealed that ceramic materials containing metal (conducting) oxides are important in enhancing the structural, morphological, and electrical properties of ZnAl_2_O_4_. Likewise, various researchers reported the use of doped ZnAl_2_O_4_ in a variety of optical and catalytic applications, including Zn(1 − *x*)Mn*x*Al_2_O_4_, Sr(II): ZnAl_2_O_4_, ZnAl_2_O_4_:Eu^3+^, and ZnAl_2_O_4_:TR (TR = Eu^3+^, Tb^3+^)^19,24–26^. Thirumanathan et al. reported the study of the formation of bismuth titanate nanoparticles by the combustion process^27^. They performed the dielectric properties of prepared nanoparticles and fabricated the patch antenna. They observed the dielectric permittivity value of 450, the dielectric loss of 0.98, and antenna’s return loss of − 4.95 dB at the resonant frequency of 2.45 GHz. In another work, Rahman et al. studied the properties of patch antenna using the sol–gel derived gahnite (ZnAl_2_O_4_) nanoparticles^28^. The obtained dielectric constant, optical bandgap, and quality factor were 8.7, 4.08 eV, and 4592, respectively. The prepared microstrip antenna demonstrated the antenna's return loss of − 25.4 dB at the resonant frequency of 12.78 GHz with its bandwidth of 760 MHz and 8.1 GHz in the low- and high-frequency bands, respectively. Abdullah et al. examined the performance of a patch antenna based on magnesium doped ZnAl_2_O_4_ ceramic nanoparticles. By varying the concentration of magnesium doping, they reported the crystallite size, lattice parameter, dielectric permittivity, return loss, and bandwidth in their range from 19.2 to 12.9 nm, 8.082 to 8.048, − 16.34 to − 21.38 dB, and 90 to 225 MHz, respectively. They fabricated the GPS patch antenna and noticed its resonant frequency at 1.570 GHz^29^.

This paper reports the synthesis and investigation of ZnAl_2_O_4_TiO_2_ dielectric ceramic nanoparticles prepared by an in-expensive and straightforward sol–gel route. Further, we employed these nanoparticles to fabricate a prototype microstrip patch antenna, which demonstrated its return loss of − 30.72 dB at a resonant frequency of 4.85 GHz. To the best of our knowledge, no similar work of fabricating a microstrip patch antenna using ZnAl_2_O_4_TiO_2_ has been reported for the C-band applications. “Materials and methods” section presents the materials and methods of synthesizing ZnAl_2_O_4_TiO_2_ composite nanoparticles. The characteristics of ZnAl_2_O_4_TiO_2_ nanoparticles, dielectric properties, and microstrip patch antenna performance are discussed in “Results and discussion” section. Lastly, “Conclusions” section summarizes the paper.

## Materials and methods

For the sol–gel synthesis of ZnAl_2_O_4_TiO_2_(ZAT) dielectric ceramic nanoparticles, titanium tetra isopropoxide (TTIP, Sigma Aldrich), zinc acetate (CH_3_COO)_2_Zn_2_H_2_O (Lobychem), aluminum nitrate nonahydrate (Al_2_(NO_3_)_3_·9H_2_O, (Sigma Aldrich), ethanol (C_2_H_5_OH, Sigma Aldrich), ethylene glycol (EG, SdFine) and nitric acid (HNO_3_, Lobychem) were procured and used without any further purification.

**Figure 1:**
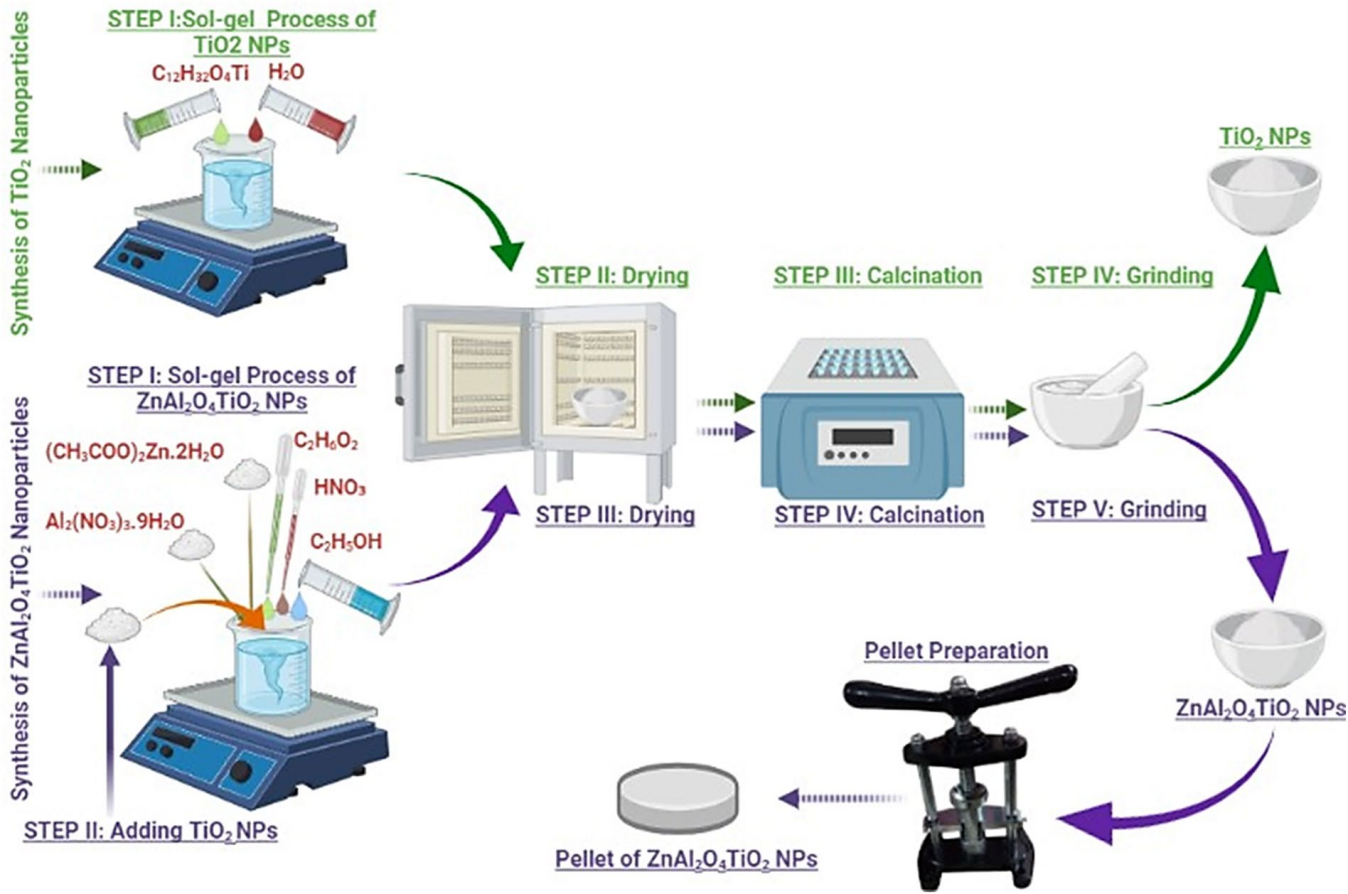
Illustration of the sol–gel synthesis process of TiO_2_ and composite ZnAl_2_O_4_TiO_2_ nanoparticles till the preparation of pellet for dielectric measurement.

Figure 1 illustrates the step-by-step process followed in synthesizing the composite ZnAl_2_O_4_TiO_2_ nanoparticles and the pellet preparation for the measurement of dielectric properties. Initially, 5 ml distilled water was added to 75 ml ethanol and stirred. Then 4.5 ml TTIP solution was added and stirred for about 4 h at approximately 85 °C. Followed by this, a white powder was obtained, calcined at 700 °C and ground. In a separate beaker, 15 g aluminium nitrate nonahydrate was added in 40 ml ethanol, and later 0.4 ml ethylene glycol (EG) was mixed in the above solution under constant stirring conditions. Finally, 6.6 g zinc acetate dehydrate and 0.32 g TiO_2_ powder (previously prepared) were mixed in sequence to the solution mentioned above at temperature 75 °C and stirred for 1 h.

During this process, 0.24 ml nitric acid (HNO_3_) was dropped into the solution for preparing the homogeneous solution and stirred at temperature 75 °C for another 1 h till the formation of a clear solution. Doing so, (1 − *x*)ZnAl_2_O_4_ − *x*TiO_2_ powder was obtained while *x* is the concentration of the TiO_2_ (i.e. *x* = 0.1). The sample was dried in an oven for 30 min at a temperature of 180 °C. Lastly, the sample was calcined at a temperature of 700 °C for 1 h and then ground. The prepared nanoparticles were employed to prepare a pellet using the pellet press machine. The prepared pellet was 1.12 mm thick and 10 mm in diameter. Prior to dielectric measurement, the as-prepared pellet was thermally treated at temperature 700 °C for 1 h and then it’s both sides were coated using the silver paste by doctor blade process.

The prepared sample named as ZA was characterized by using an X-ray diffractometer (XRD, X-Pert Pro, UK), Raman spectroscopy (BWTEK, Japan), Transmission electron microscopy (TEM, TALOS F200S G2, USA), energy dispersive X-ray spectroscopy (EDS), and LCR meter (PSM1735 N4L, Newtons4th Ltd, UK). The fabricated prototype microstrip patch antenna based on composite nanoparticles was tested using the Vector Network Analyzer (VNA, R&S®ZVL, Germany).

## Results and discussion

**Figure 2:**
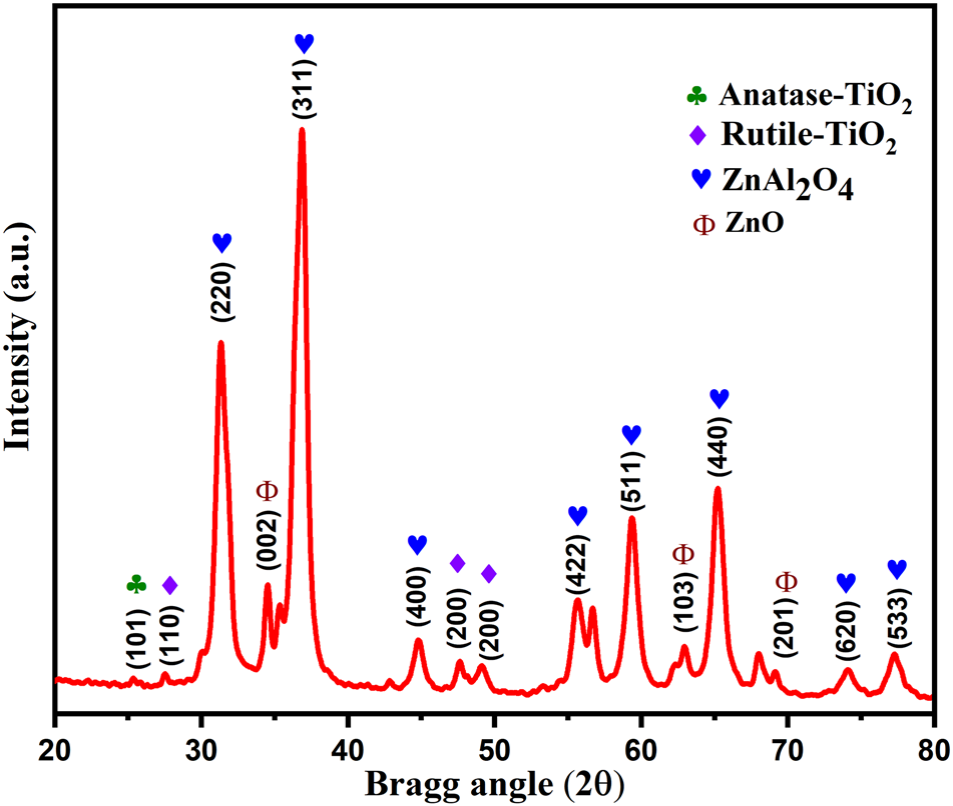
XRD pattern of ZnAl_2_O_4_TiO_2_ dielectric ceramic nanoparticles prepared by the sol–gel method.

The X-ray diffraction (XRD) investigation is advantageous for determining the crystalline phases of the nanomaterials. Figure 2 depicts the XRD pattern of ZAT nanocomposite dielectric ceramic material. It represents the various peaks of ZnAl_2_O_4_ crystal structure corresponding to the typical face-centered cubic morphology and was found consistent with the reported literature^30,31^. One can also notice the formation of the crystalline structure of titanium dioxide (TiO_2_) with its anatase and rutile phases and the wurtzite structure of ZnO^32,33^. Our XRD result also coincides with the JCPDS File No. 00-021-1272 and 01-021-1276 of anatase and rutile TiO_2,_ respectively. Additionally, it matches well with the JCPDS File No. 00-005-0669 and 89-0510 corresponding to the ZnAl_2_O_4_ and ZnO. Compared to the pristine ZnAl_2_O_4_ sample, which showed the various peaks of ZnAl_2_O_4_, some peaks of ZnO can also be noticed^18,19^. Further, in the composite sample of ZnAl_2_O_4_TiO_2_ the additional peaks of TiO_2_ were noticed^34,35^. In addition, the peak locations were found slightly shifted with the increased value of TiO_2_ concentration. In other words, the unit cell dimension was noticed to be decreased with the enhanced crystallinity^34,36^. In our case, the crystallite size was estimated to be 13.3 nm using the Scherrer formula (d = 0.94λ/(βcosϴ), where β is the X-ray wavelength, and β is the full-width at half-maximum intensity of the diffraction line).

**Figure 3:**
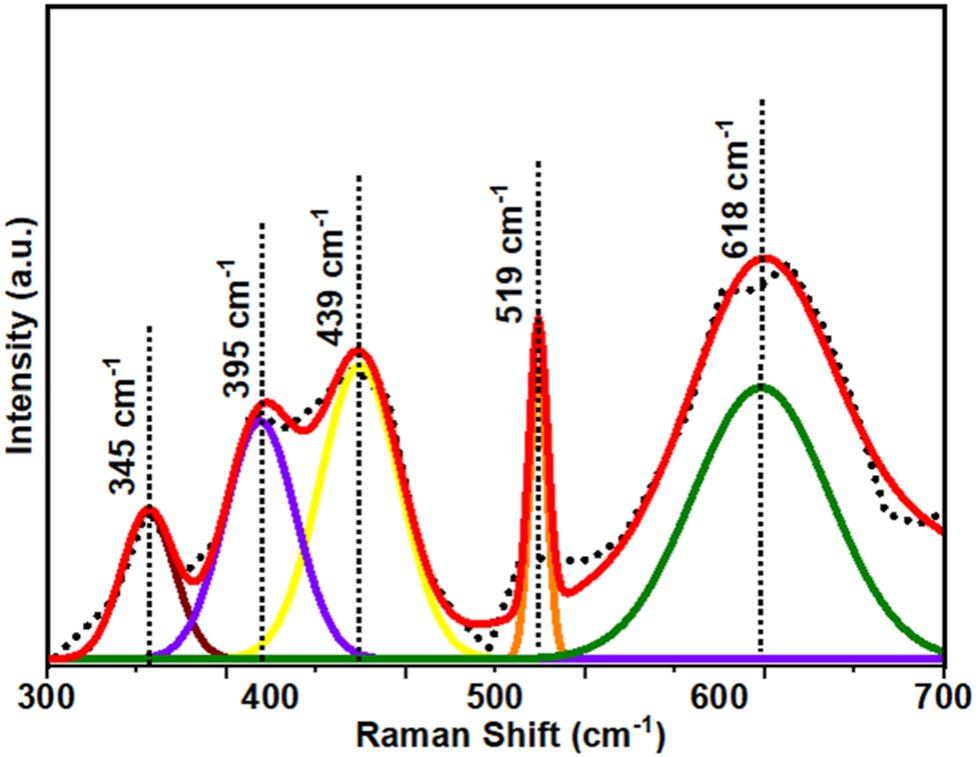
Raman spectra of ZnAl_2_O_4_TiO_2_ dielectric ceramic nanoparticles.

Figure 3 shows the Raman spectra of ZnAl_2_O_4_TiO_2_ nanoparticles sintered at a temperature of 700 °C. We noticed various peaks from the prepared sample that originated due to ZnAl_2_O_4_, ZnO, and TiO_2_ contents. We can observe two Raman peaks originated at 395 cm^−1^ and 519 cm^−1^ assigned to B1g and A1g/B1g modes, respectively, showing the impression of anatase-TiO_2_. Further, a peak 439 cm^−1^ known as E_2_ high vibration mode was associated with oxygen atoms and assigned to ZnTiO_3_ nanocrystals. A broad peak at 618 cm^−1^ represents the thermodynamically stable rutile-TiO_2_ relates the space group D_4h_ assuming the site symmetries for the Ti and O atoms within the unit cell. In literature, this peak is attributed to the Raman-active “lattice vibration” designated as A_1g_^37–39^.

**Figure 4:**
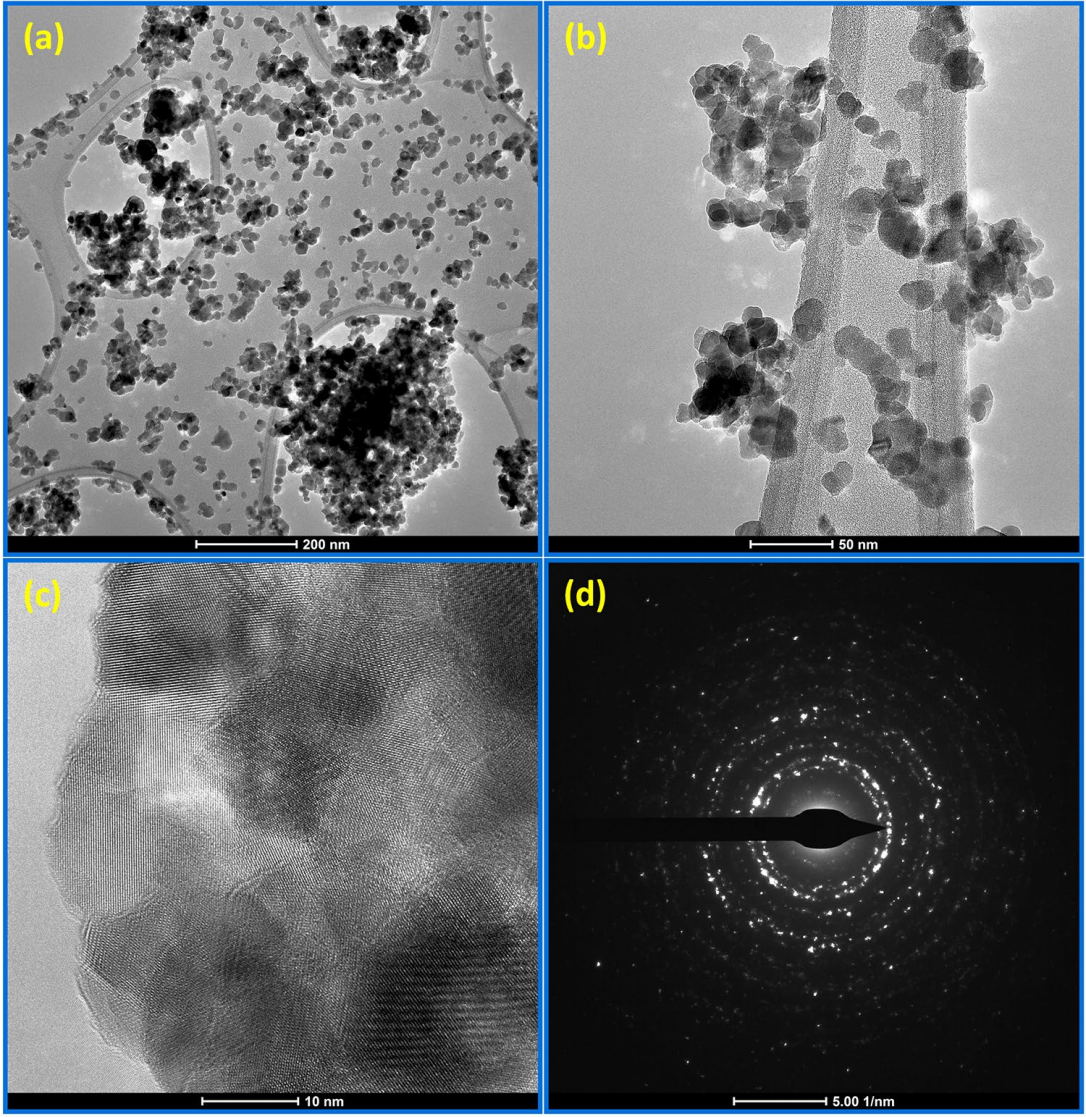
TEM micrographs at scale 200 nm (**a**) at scale 50 nm (**b**), HRTEM image (**c**) and SAED pattern (**d**) of ZnAl_2_O_4_TiO_2_ dielectric ceramic nanoparticles.

TEM measurement was carried out to know the morphology of ZAT dielectric ceramic nanoparticles. Figure 4a depicts the good yield of ZnAl_2_O_4_TiO_2_ nanoparticles as recorded at the scale of 200 nm. We can observe almost well-dispersed nanoparticles with very low agglomeration in the TEM micrograph as shown in Fig. 4b, which was recorded at the scale of 50 nm. Figure 4c depicts the high-resolution TEM micrograph of ZAT nanoparticles. The calculated inter-spacing (d) values were 0.130, 0.145, and 0.167 nm, corresponding to the reflection of the planes of (201), (103), and (110). The selected-area electron diffraction (SAED) pattern depicted in Fig. 4d depicts the appearance of solid rings representing the polycrystalline nature of the ZAT nanoparticles. The result coincides with the XRD pattern as discussed in Fig. 2.

**Figure 5:**
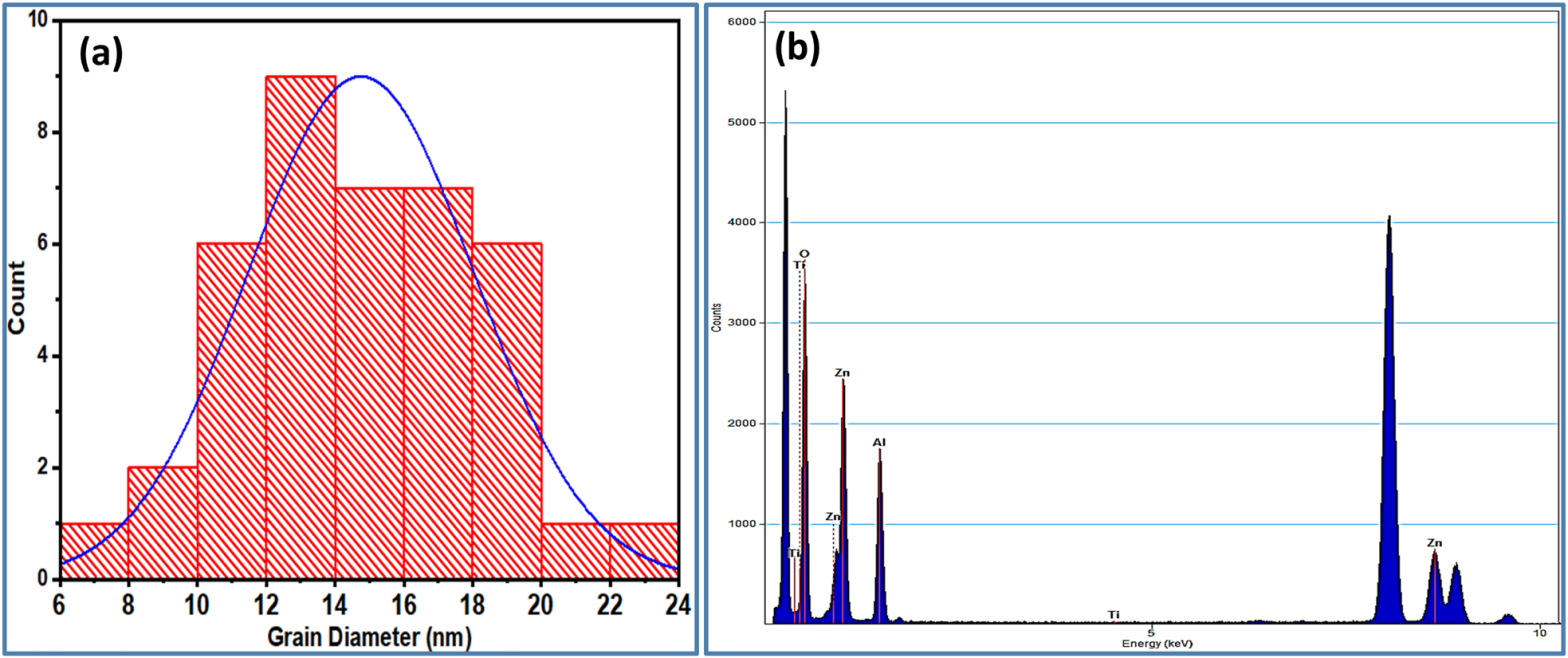
Histogram (**a**) and EDS spectrum (**b**) of ZnAl_2_O_4_TiO_2_ dielectric ceramic nanoparticles.

Figure 5a illustrates the histogram of ZnAl_2_O_4_TiO_2_ nanoparticles by measuring the diameters of about 40 grains to estimate the average size. As can be seen, the dielectric ceramic nanoparticles have an average diameter of 14.75 nm.

The EDS spectrum of ZnAl_2_O_4_TiO_2_ dielectric ceramic nanoparticles is shown in Fig. 5b. The EDS spectrum revealed the elemental peaks of O, Zn, Al, and Ti at energy values 0.52, 1.11, 1.48, and 4.5 keV, respectively.

The dielectric permittivity value represents the material’s ability to store electric energy when an electric field is applied, and it is related to the capacitance associated with the dipole orientation of charge carriers. After obtaining the parallel capacitance values, we have calculated the dielectric permittivity by using an expression, ${\varepsilon }_{r}=Cd/{\varepsilon }_{o}A$, where C is the capacitor’s capacitance, d is the pellet’s thickness, *ε*_0_ is the permittivity of free space, and A is the cross-section area of the pellet. The dielectric characteristic of ZnAl_2_O_4_TiO_2_ nanoparticles was studied using the LCR meter with its frequency range from 100 Hz to 1 MHz at room temperature.

**Figure 6:**
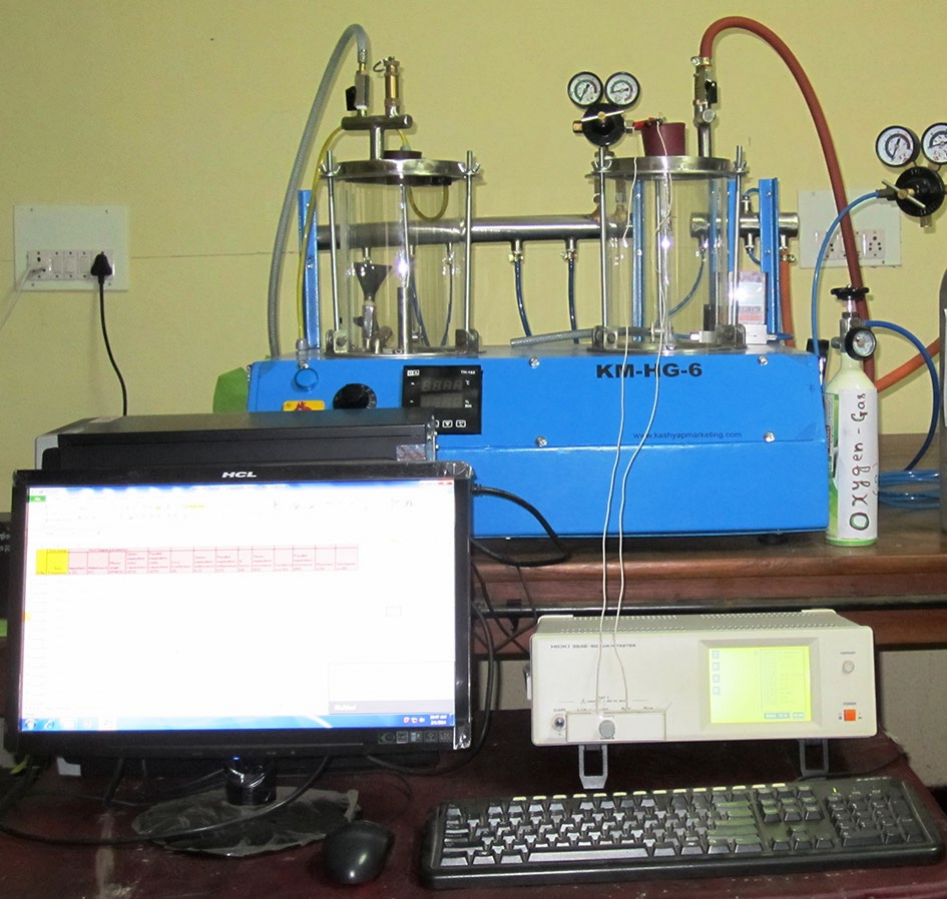
The setup of dielectric measurement.

Figure 6 shows the dielectric measurement setup used in this study. The LCR meter is connected to the computer, while the front panel (bottom-left) of the LCR meter is loaded with a pellet under the test. One can also do the dielectric measurement by varying the temperature through a separate unit, as shown here. However, the dielectric measurement was carried out at room temperature in this case.

**Figure 7:**
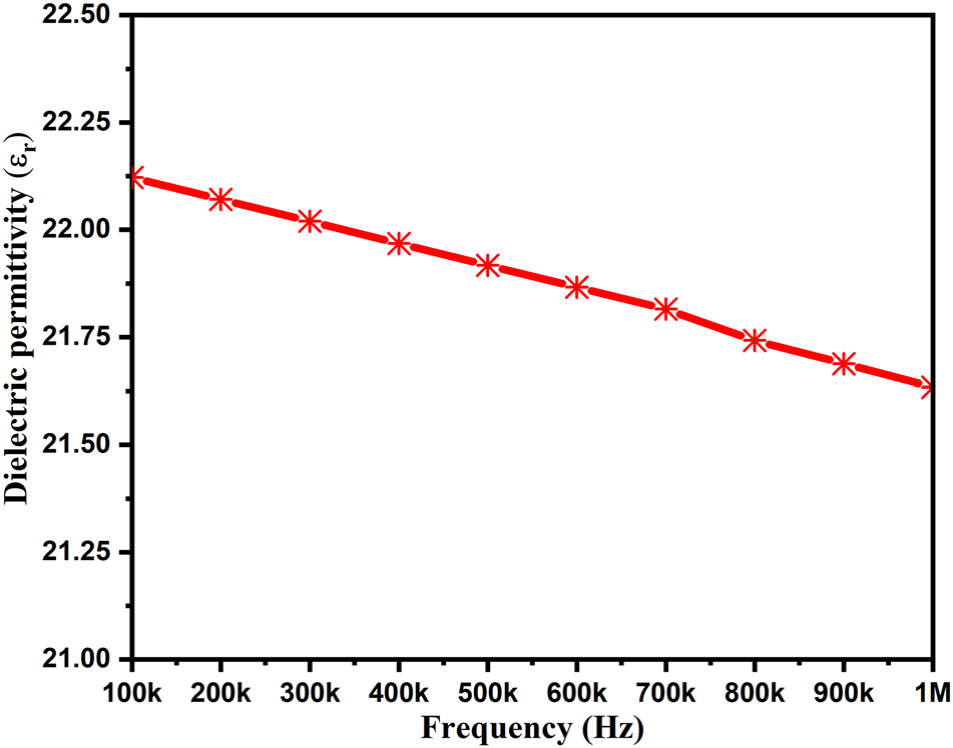
Dielectric permittivity of ZnAl_2_O_4_TiO_2_ dielectric ceramic nanoparticles.

Figure 7 depicts the variation of dielectric permittivity in accordance with the frequency. With the increased frequency, one can observe the decreased permittivity. The dielectric permittivity was varied from 22.12 to 21.63 with an increased frequency range from 100 kHz to 1 MHz. Roshini et al. also reported the ZnAl_2_O_4_TiO_2_ material with its dielectric constant of 9.6^40^. Similarly, Abdullah et al. studied the dielectric permittivity of ZnAl_2_O_4_-SiO_2_ nanoparticles and reported its value of 8.57^20^. An abrupt decrease in dielectric permittivity in the lower frequency range was noticed, which was found constant in the higher frequency region. This typical characteristic of such materials can be attributed to the reduced polarization^39^.

**Figure 8:**
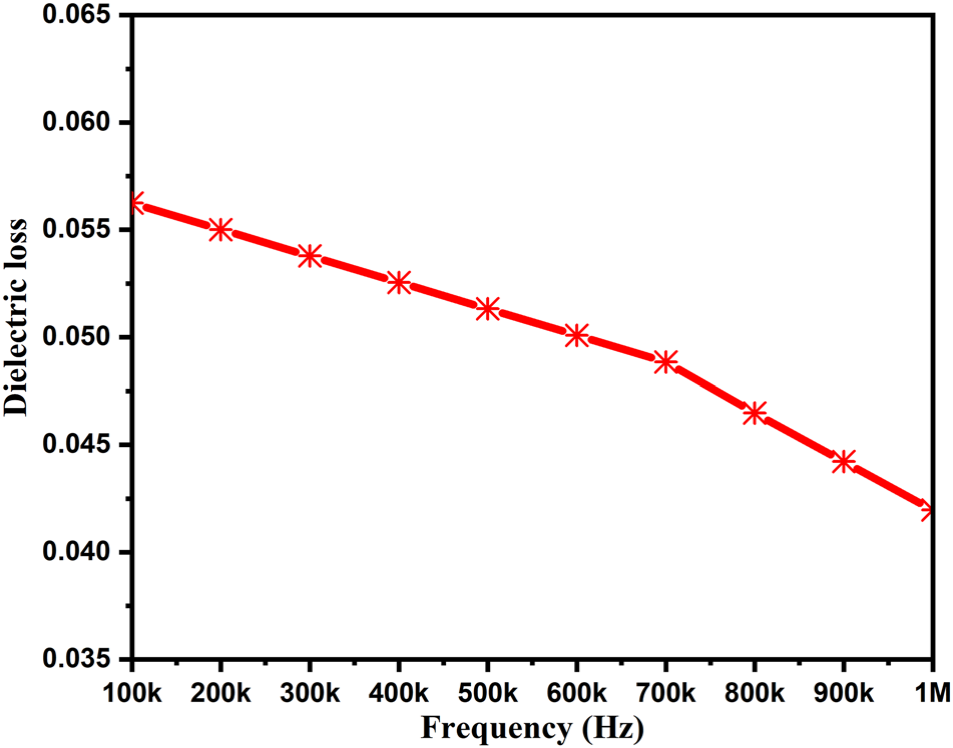
Dielectric loss of ZnAl_2_O_4_TiO_2_ dielectric ceramic nanoparticles.

Dielectric loss (tanδ) is an important parameter representing the energy dissipation, and therefore, it needs to be studied. Dielectric loss is also regarded the microstructure faults, e.g. microstructural defects, porosity, micro-crashes, the spontaneous orientation of crystallite, etc.^21^. Figure 8 depicts the dielectric loss of ZnAl_2_O_4_TiO_2_ nanocomposite ceramic sample as a function of frequency. The dielectric loss was noticed to be decreased from 0.056 to 0.041 with an increased frequency range from 100 kHz to 1 MHz. In general, one can observe the reduced dielectric loss with the increased frequency. This nature is because the hopping ions lag behind the applied electric field. At the lower frequency range, one can notice the increased dielectric loss value, which further decreases in the higher frequency region. In the first case, this relates to the high resistivity resulting from the associated effect of grain boundaries.

**Figure 9:**
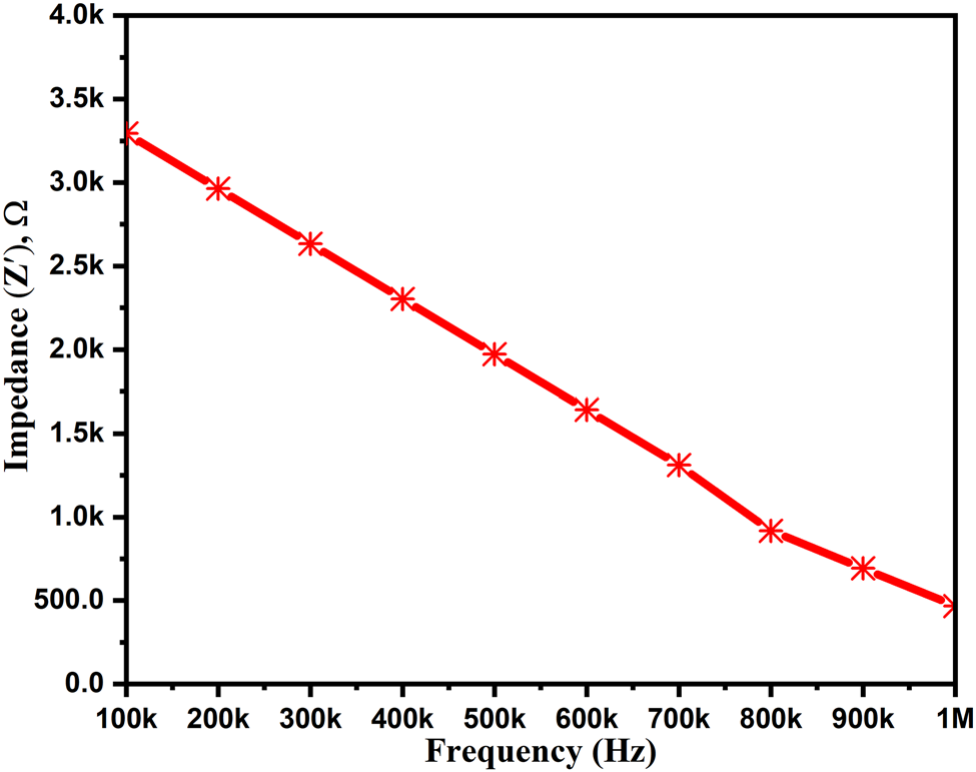
Impedance variation (real part) of ZnAl_2_O_4_TiO_2_ dielectric ceramic nanoparticles.

**Figure 10:**
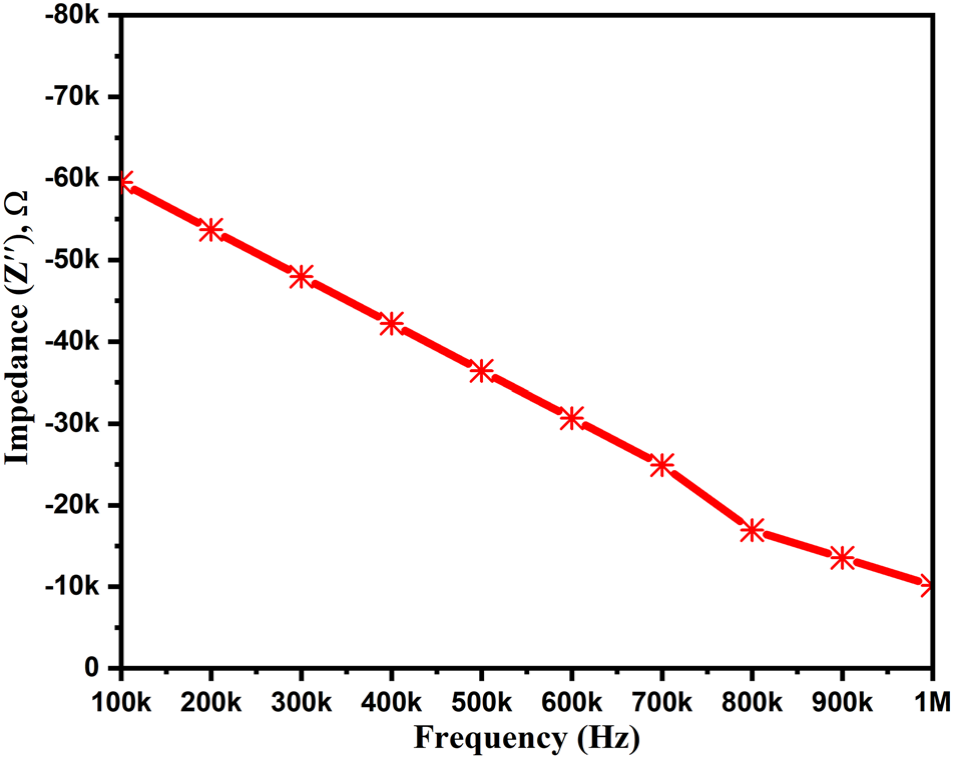
Impedance variation (imaginary part) of ZnAl_2_O_4_TiO_2_ dielectric ceramic nanoparticles.

We have calculated the real (Z′) and imaginary (Z″) impedance values with respect to the frequency, which are plotted in Figs. 9 and 10, respectively. As shown in Fig. 9, we can notice the decreased real-impedance from 3.28 kΩ to 467 Ω with the rise in frequency. Similarly, Fig. 10 depicts the same trend of increased imaginary impedance values from − 59 to − 10 kΩ with the increased frequency from 100 kHz to 1 MHz. The Z′ value fluctuates with temperature and joins together in the higher frequency region (not shown here). This happens due to the liberation of charge carriers and semiconducting characteristics at high temperatures^40^. However, the Z′′ value rises with temperature, and the larger value at a higher frequency regime indicates the increase in tangent loss.

**Figure 11:**
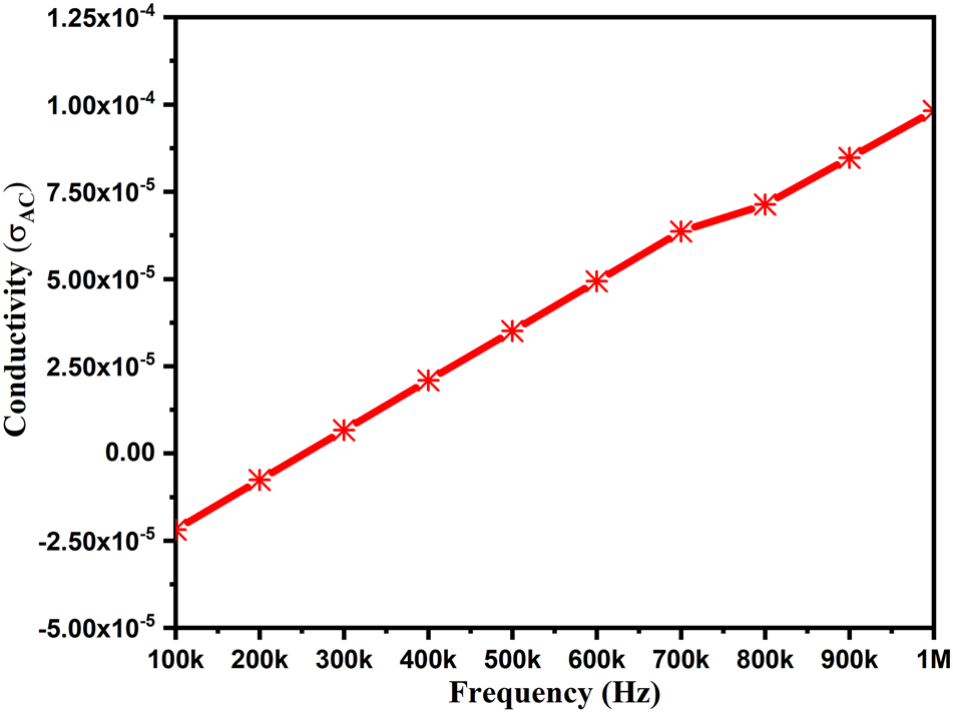
Conductivity of ZnAl_2_O_4_TiO_2_ dielectric ceramic nanoparticles.

Figure 11 illustrates the frequency-dependent ac conductivity at room temperature. The ac conductivity was estimated using the relation σ_ac_ = ωεε_o_tanδ, where ε_o_ is the free space dielectric permittivity, ε is dielectric permittivity, ω is the angular frequency, and tanδ is the tangent loss. The investigation of conductivity as a function of frequency relates to the process of charge transport. We can notice the enhancement in conductivity from 2.2 × 10^–5^ to 9.8 × 10^–5^ with the increased frequency. This increasing trend of conductivity in the lower frequency range (not shown here) can be attributed to space charges scattering cations across adjacent sites^41^. The conductivity curve coincides at high-frequency band, representing that the conductivity curves obey Jonscher’s power law and therefore exhibit low-frequency dispersion phenomena^42^.

**Figure 12:**
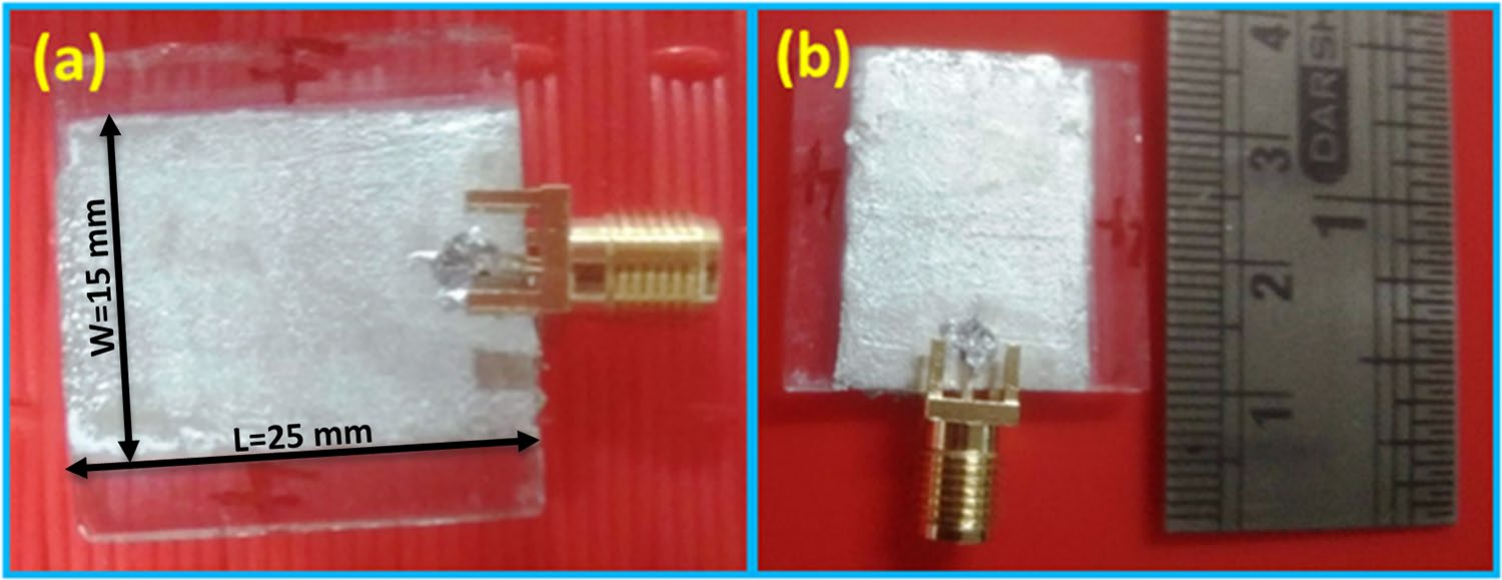
Digital images of the prototype microstrip patch antenna.

The prepared ceramic dielectric ZnAl_2_O_4_TiO_2_ nanoparticles were employed for preparing a patch antenna. Initially, ZAT paste was prepared, which was cast on the FTO substrate and then it was silver coated on both sides for metal contacts. Finally, the SMA connector was connected to it, and antenna performance was evaluated using a vector network analyzer. Figure 12 depicts the patch antenna’s top view which illustrates its dimension and shape. The fabricated prototype microstrip patch antenna has its length and width of 25 mm and 15 mm, respectively, as illustrated in Fig. 12a.

**Figure 13:**
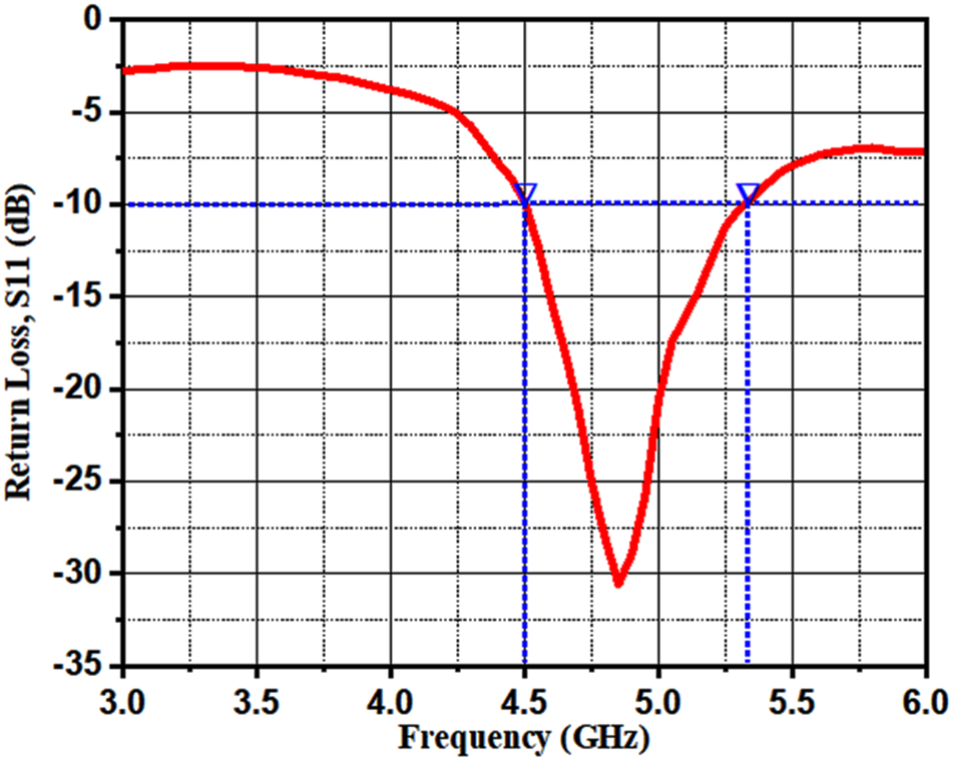
Return loss of microstrip patch antenna based on ZnAl_2_O_4_TiO_2_ nanoparticles.

Figure 13 depicts the measured return loss of ZnAl_2_O_4_TiO_2_ microstrip patch antenna measured in the range from 4 to 6 GHz. The fabricated antenna covered the minimum required value of return loss, i.e. − 10 dB. The prototype microstrip antenna demonstrated a resonant frequency of 4.85 GHz and the return loss of − 30.72 dB with its bandwidth of 830 MHz.

## Conclusions

Dielectric ceramic ZnAl_2_O_4_TiO_2_ nanoparticles prepared using the low-cost and easy technique have been studied. The synthesized nanoparticles were crystalline with their crystallite size of 13.3 nm. The Raman study evidenced the corresponding Raman shift of the constituent elements presented in the composite nanoparticles. The morphological investigation of the nanoparticles endorsed the formation of spherical grains with their mean diameter of 14.75 nm. The crystallinity of the prepared sample studied by the SAED pattern was consistent with the XRD result. The LCR meter measurement showed the decreased dielectric permittivity and loss as a function of applied frequency. The performance of the prototype microstrip patch antenna based on the dielectric ceramic nanoparticles was also studied. The microstrip patch antenna exhibited its return loss of − 30.72 dB at the resonant frequency of 4.85 GHz with its bandwidth of 830 MHz. In summary, a microstrip patch antenna demonstrated its resonant frequency in the C-band range, which lies from 4 to 8 GHz, and therefore, this material would be suitable for satellite communications, weather radar systems, terrestrial microwave links, and 802.11 versions of Wifi devices.
